# Comparative physiological and metabolomics analysis of wheat (*Triticum aestivum* L.) following post-anthesis heat stress

**DOI:** 10.1371/journal.pone.0197919

**Published:** 2018-06-13

**Authors:** Kayla Thomason, Md Ali Babar, John E. Erickson, Michael Mulvaney, Chris Beecher, Greg MacDonald

**Affiliations:** 1 Agronomy Dept., University of Florida, Gainesville, FL, United States of America; 2 West Florida Research and Education Center, University of Florida, Jay, FL, United States of America; 3 Southeast Center for Integrated Metabolomics (SECIM), University of Florida, Gainesville, FL, United States of America; 4 IROA Technologies LLC, Ann Arbor, MI, United States of America; Institute of Genetics and Developmental Biology Chinese Academy of Sciences, CHINA

## Abstract

Genetic improvement for stress tolerance requires a solid understanding of biochemical processes involved with different physiological mechanisms and their relationships with different traits. The objective of this study was to demonstrate genetic variability in altered metabolic levels in a panel of six wheat genotypes in contrasting temperature regimes, and to quantify the correlation between those metabolites with different traits. In a controlled environment experiment, heat stress (35:28 ± 0.08°C) was initiated 10 days after anthesis. Flag leaves were collected 10 days after heat treatment to employ an untargeted metabolomics profiling using LC-HRMS based technique called IROA. High temperature stress produced significant genetic variations for cell and thylakoid membrane damage, and yield related traits. 64 known metabolites accumulated 1.5 fold of higher or lower due to high temperature stress. In general, metabolites that increased the most under heat stress (L-tryptophan, pipecolate) showed negative correlation with different traits. Contrary, the metabolites that decreased the most under heat stress (drummondol, anthranilate) showed positive correlation with the traits. Aminoacyl-tRNA biosysnthesis and plant secondary metabolite biosynthesis pathways were most impacted by high temperature stress. The robustness of metabolic change and their relationship with phenotypes renders those metabolites as potential bio-markers for genetic improvement.

## Introduction

Wheat (*Triticum aestivum* L.) is grown globally and is one of the most important food crops for food security. A significant challenge to wheat production and optimal yields is high temperature stress [1], which will be exacerbated with expected increases in temperature associated with global climate change [2]. It has been shown that global wheat production is already declining due to warming and that with each 1°C increase in temperature it is estimated that global wheat production will decline by 6% [2]. The negative effects of increased temperatures on wheat yield and production have led to research to understand the effects of high temperature stress on wheat physiology and breeding for genotypes better able to cope with high temperature stress [3]. Identifying cultivars with high yield potential under stress environments will be critical to improve the productivity of wheat under expected future climatic conditions. Over the long-term, genetic improvement is potentially the most effective and sustainable method to achieve this goal. However, genetic improvement requires a solid understanding of the biochemical mechanisms controlling different traits. Significant breeding efforts have achieved some genetic improvement for stress tolerance, but an understanding of the metabolites associated with the traits that confer heat tolerance in wheat, could contribute to an improved understanding of heat tolerance and for germplasm improvement.

Anthropogenic activities, such as land use change and combustion of fossil fuels, have resulted in the release of carbon dioxide and other greenhouse gases (GHG) that have been linked to increasing climate variability and global climate change [4]. In the northern hemisphere, the period of time from 1983 to 2012 was likely the warmest 30-year period in the last 1400 years with an estimate of approximately a 1°C increase in global surface temperature from 1880 to 2012 [4]. Current and continued emissions of GHG are expected to cause further warming, especially at higher latitudes [4]. Since wheat is predominately grown at higher latitudes, this is of particular concern for wheat production. With temperatures becoming warmer in spring and early summer, wheat plants may be subject to increased terminal heat stress [5].

Wheat is more sensitive to heat stress during the reproductive phase than the vegetative stage [6]. Heat stress imposed immediately prior to and during anthesis results in pollen sterility, which reduces grain number. Experimental evidence found that exposing wheat plants to temperatures of 36/31°C (day/night) from head emergence until 10 days after anthesis resulted in reduced yields from grain sterility [7]. Temperatures greater than 30°C can cause complete sterility during floret formation [5]. High temperature during the grain-filling period decreases yield by decreasing kernel weight [8]. Kernel weight was decreased by 85% when the temperature rose from 20/16°C to 36/31°C from 7 days after anthesis until maturity [9]. In the hard red winter wheat Karl 92, grain yield was reduced by 78%, kernel number by 63%, and kernel weight by 29% when a temperature regime of 35/20°C was imposed from 10 days after anthesis until maturity [10]. In addition, grain yield is more sensitive to increased night temperatures than daytime temperatures. Photosynthesis is reduced during heat stress due to a reduction in chlorophyll content and an increase in oxidative stress [11; 12]. In wheat, optimal rates of photosynthesis are around 25°C and decrease above 30°C [6]. While plants are under heat stress and assimilation declines, stem reserve mobilization can become an important source for grain yield [13]. Heat stress accelerates plant senescence and this reduction in growth duration is a major cause of decreased yield [14].

Although measuring crop physiological and morphological parameters can be informative, it often does not provide a picture of their underlying relation with changes of metabolite status in plants under stress conditions. Recent advances in mass spectrometry, especially bioinformatics, allows for comprehensive and comparative metabolic profiles on crop genotypes [15; 16; 17]. Plants produce an array of metabolites, which can differ under stress conditions. Differences in metabolite expression are associated with different genotypic and phenotypic traits [18]. Thus, metabolomics could be a powerful selection tool to establish the association between phenotype and genotype, leading to a better understanding of the genetic basis of plant responses to stress. For example, recent studies have demonstrated that metabolite expression differed between drought-stressed and well-watered plants [16; 17; 19; 20; 21; 22]. The expression level of branched chain amino acids (leucine, isoleucine, and valine) was increased under drought in tolerant cultivars of wheat [20; 23; 24] and barley (Hordeum vulgare L.) [25]. Differing metabolite expression was associated with high temperature stress tolerance in maize (Zea mays L.) [26] and in cool-season grasses [27]. Maize plants under high temperature stress showed increases in metabolites such as tryptophan, serine, threonine, beta-alanine, proline, glutamate, myo-inositol, and urea [17]. In addition to this, certain metabolites showed a negative correlation (threonine, valine, trehalose, glycerol) or a positive correlation (fumarate, succinate, raffinose) with grain yield, and that metabolic expression was consistent for greenhouse plants and field-grown plants [17].

Plant biologists are still facing challenges in linking metabolite expression to the biochemical mechanisms involved in stress tolerance, and to identify suitable breeding strategies to use that information in specific selection programs. Thus, identifying associations between metabolites and different physiological and yield related traits under stress provides a novel opportunity for plant breeders. Comparative metabolomics studies can link plant function to metabolite status, which can enhance understanding of heat stress tolerance in wheat and could offer a set of related metabolome biomarkers. If heat stress related metabolic biomarkers can be identified for wheat, they could be used as targeted and fast diagnostic tools to select germplasm with improved performance under higher temperatures. At present, there are insufficient biomarkers available to adequately and efficiently screen cereal crops for heat stress tolerance or susceptibility. The objectives of this research were to 1) quantify the effects of post-anthesis heat stress on wheat physiology and yield; 2) compare metabolite profiles of wheat under control and post-anthesis heat stress; and 3) identify correlations between wheat performance and metabolite expression under heat stress, and relate those metabolites to different biochemical pathways in the plant. To achieve these objectives, we employed a non-targeted LC–MS Isotopic Ratio Outlier Analysis (IROA) Global Metabolomics method [28] for identifying metabolites from the leaf tissue of optimal (24:18 ± 0.08°C) and post-anthesis, heat-stressed (35:28 ± 0.08°C) wheat plants.

## Materials and methods

### Genetic materials, growth and experimental conditions

Six wheat genotypes were used for the study, which included Ventnor, CB208 (VA12W72), CB210 (LA03200E2), CB213 (LA06027EP7), CB205 (AGS2000), and CB202 (USG3120). The study was conducted in 2014–15 and repeated again in 2015–16. These genotypes represent a broad genetic base of soft red wheat germplasm grown in the southeastern USA. AGS2000 (PI612956; PIO2555/PF84301//Florida302) was developed and released jointly by the University of Georgia and University of Florida [29]. USG3120 (PI672163; GA901146/GA9006//AGS2000) is a medium maturing soft red winter wheat with white chaffed and medium in height. Both AGS200 and USG3120 are early maturing high yield soft red wheat varieties which are grown commercially throughout South and Southeastern USA. CB208 (VA12W72), CB210 (LA03200E2), and CB213 (LA06027EP7) are advanced soft wheat breeding lines developed by wheat breeding programs of Virginia Tech and Louisiana State University. The advanced soft wheat breeding lines and varieties were selected for the study based on the stay green and yield related traits under field conditions in 2013–14 in Quincy, FL, where temperatures between 30 and 36°C are common during the reproductive stage of wheat. In the field evaluation, CB202 and CB208 showed longer stay green trait than other three genotypes, and were considered as heat tolerant genotypes. In a follow up study under greenhouse conditions, those two genotypes demonstrated superior stay green capacity compared to the check wheat line (Ventnor) under high temperature stress. Ventnor is an Australian spring wheat variety (unknown pedigree) and has been shown to maintain photosynthetic capacity and kernel weight when exposed to post-anthesis heat stress [30; 31].

Initially, the seeds of the genotypes were exposed to vernalization at 4°C for 6 weeks. After 6 weeks of vernalization, germinated seeds were transplanted to pots filled with Metro-Mix (Sun Gro Horticulture, Agawam, MA, USA) artificial media and grown in a climate controlled polycarbonate greenhouse facility located in Gainesville, Florida. Each pot contained three plants and a total of six pots per genotype were used in the study for a total of 18 plants in each year. The pots were well watered throughout the growth cycle and a teaspoon of a blended granular slow-release fertilizer (15-9-12) was applied one time after transplanting. The growing conditions were maintained at a day:night temperature of 20:15 ± 0.08°C, relative humidity of 60 ± 0.3%, and a 16:8 day:night photoperiod. The temperature treatments were initiated ten days after anthesis (determined when over 50% of the tillers were flowering). Three control pots per genotype were kept at a day:night temperature of 24:18 ± 0.08°C, relative humidity of 60 ± 0.3%, 16 h photoperiod using supplemental light from four 400-W metal halide lamps, while the heat-stressed plants were exposed to a day:night temperature of 35:28±0.08°C, under the same humidity, light, and day length conditions.

### Measures of physiological traits

Data were collected on physiological parameters known to be affected by heat stress in wheat [3]. In 2014/15, measurements were taken at 7 and 14 days after temperature treatment initiation. In 2015/16, we took measurements at 7, 10, and 14 days after treatment initiation due to how quickly the plants senesced in the first experiment. All measurements were taken on the flag leaf of each plant. Estimates of leaf chlorophyll content were taken using a SPAD chlorophyll meter (Model 502, Spectrum Technologies, Plainfield, IL, USA). Three measurements were taken per leaf and averaged (9 measurements per pot as each pot had three plants). The average value of 9 measurements was used as one replication during statistical analysis. The relative damage to SPAD chlorophyll content (SCC) due to heat stress was assessed by comparing SPAD values between control and heat treated plants. The relative damage to chlorophyll content was estimated as: % SCC = [(SPAD _control_-SPAD _heat_)/SPAD _control_]*100 by using average across years due similar results for each experiment. An OS30p+ modulated fluorometer (Opti-Sciences, Hudson, NH, USA) was used to measure dark-adapted F_V_/F_M_. To insure dark adaption, opaque clips were put on the leaves thirty minutes before F_V_/F_M_ measurements were taken during middle of the day. Chlorophyll fluorescence, the ratio of variable (F_V_) to maximum fluorescence (F_M_), was used as an indirect method to assess thylakoid membrane damage [32; 33]. To assess damage to thylakoid membrane (TMD) due to heat stress, we compared F_V_/F_M_ values between control and heat-treated plants. The relative damage was estimated as: % TMD = [(Fv/Fm _control_—F_V_/F_M heat_)/ F_V_/F_M control_]*100 by using average across years due similar results for each experiment. Cell membrane stability (CMS) was measured by adapting the method of [34]. Six, 5-mm leaf punches were taken per flag leaf, placed into 24 mL of DI water (4 mL per leaf punch), vortexed, and put on a shaker for 24 hours at approx. 4°C. After 24 hours, the samples were placed on a shaker at room temperature for one hour, vortexed, and then the electrical conductivity (EC) of the solution was measured using an accumet epoxy 4-band conductivity cell EC probe (Fisher Scientific International Inc., Pittsburgh, PA, USA) with a 1 cm^-1^ nominal cell constant. The samples were then autoclaved for 15 minutes to lyse the tissue cells and release the remaining electrolytes. After autoclaving, the samples were placed on a shaker at room temperature for one hour, vortexed, and EC was measured again. CMS (% damage) was then calculated according to the equation by [35]: 100 × (% leached _heat_—% leached _control_)/(X—% leached _control_), where ‘X’ was the % leached value corresponding to 100% damage, which was assumed to be 100% leached. We used CMS values averaged across years due similar results for each experiment. As the genotypes showed similar trend for reducing in thylakoid membrane, cell membrane stability and chlorophyll content due high temperature stress in both years, we pooled data of both years for statistical analysis.

### Measures of growth and yield related traits

Plants were harvested after physiological maturity (when plants were senesced) and partitioned into spikes and stalk. Prior to harvest, spikes/plant was counted. Plants were put into ovens at 50°C for 7–10 days. The spikes were hand threshed and the chaff was cleaned off using a seed cleaner. The straw and grain were weighed and the grain number was counted. Average individual grain weight was determined by dividing the total grain weight by the grain number. Grain yield per spike (g spike^-1^) was calculated by dividing total grain weight by spike number. Grain number per spike was calculated by dividing total grain number by the number of spike for that plant. The percent reduction to yield related traits due to heat stress was assessed by comparing values between control and heat treated plants, and was calculated as follows: % reduction = [((control values)-(heat-stressed values))/(control values)] *100.

### Leaf tissue sampling for metabolomic analyses

Flag leaves were harvested for metabolomic analyses 10 d after temperature treatments (flowering + 20 d) in 2014/15. Leaf tissue (flag leaf of main tiller) of one of the three plants was collected from each pot and was considered a biological replication, and thus triplicate samples were collected at each temperature treatment for each genotype. Sampled leaf tissues were frozen in liquid N_2_ immediately after collection and stored at -80°C. Leaf tissue samples were lyophilized and ground using a tissue lyser, and subsequently stored at -80°C. For metabolomic analysis, 5 mg of the dried experimental material was weighed and added to 5 mg of wheat internal standard, both were ground just prior to analysis. The internal standard was an isotopically labeled wheat leaf that had been grown in an atmosphere of ^13^C labeled carbon dioxide resulting in a uniform and universal labeling of approximately 97% (IROA Technologies, Bolton, MA, USA). The dried powder was combined with 500 μL of methanol/10mM aqueous ammonium acetate (50:50) and vortexed for 1 minute at room temperature. The resulting mixture was sonicated for 20 minutes, and centrifuged at 17,000 G for 10 minutes. 350 μl of the supernatant was transferred to a 1.5 mL Eppendorf tube, then dried under a gentle nitrogen gas stream at 30°C. The dried sample was reconstituted in 100 μl of 0.1% aqueous format.

### Untargeted metabolomic analyses

Reconstituted samples were analyzed on an untargeted metabolomics Liquid Chromatography High Resolution Mass Spectrometry (LC/HRMS) platform at the University of Florida Southeast Center for Integrated Metabolomics (SECIM). Untargeted metabolomics profiling was performed on a Thermo Q-Exactive Oribtrap mass spectrometer with a Dionex UHPLC and autosampler. All samples were analyzed in positive and negative modes with heated electrospray ionization with a mass resolution of 70,000 at m/z 200 as separate injections. Chromatographic separation was achieved on a ACE 18-pfp 100 x 2.1 mm, 2 μm column with mobile phase A as 0.1% formic acid in water and mobile phase B as acetonitrile, with a flow rate of 350 μL/minute with a column temperature of 25°C. Total run time per sample was 21 minutes. QA/QC guidelines were followed in untargeted profiling assays with the addition of stable-isotopic internal standards to evaluate reproducibility, injection standards, the repeated analysis of a large pooled plasma sample and neat QCs that enabled assessment of an untargeted profiling run. Injection reproducibility is typically less than 10% even without a ratio to an internal standard. The native Thermo .raw output files were converted to .mzXML files using ProteoWizard (Version 2).

### Data processing and statistical analysis

Since we used an IROA-labeled plant material as our internal standard, this study followed the IROA “Phenotypic” global labeling and bioinformatics protocols in which the Internal Standard (IS) is labeled at 95% ^13^C. Therefore, all biological compounds are paired natural abundance (NA) and IS, and each pair carries distinct molecular signatures. Molecules can be distinguished from each sample set, as they have differing masses [36; 37]. For IROA, control and heat treated samples were analyzed as a single composite sample by LC-MS. Algorithms pair identified biological peaks, and unlabeled NA artifacts were identified and discarded. All biological compounds had two paired peaks; the peak from the ^12^C-media is mirrored by a second peak from the 13C-media. The distance between the monoisotopic peaks readily identified the number of carbons in the compound. The corresponding M+1 and M−1 peaks (and M^+2^ and M^−2^ etc. peaks) which are a mass difference of 1.00335 amu (mass difference between a ^12^C and ^13^C isotope), gave the IROA peaks a characteristic U-shape “smile” pattern. Accurate mass together with the knowledge of the number of carbons in a molecule greatly facilitated metabolite identification.

The ClusterFinder program was used to identify, align and quantitate the IROA peaks in the .mzXML files. The files were scanned for IROA peaks down to an intensity level of 1 million in both positive and negative modes with an assumed maximum of 10 ppm mass error. Settings for the experimental samples in the non-targeted analysis assumed a natural abundance (1.1%) isotopic balance while the internal standard was assumed to have a 97% isotopic balance. The resulting list of IROA peaks was manually curated to define 316 compounds that were seen in both the internal standard and in the experimental samples. The original non-targeted analysis necessarily leaves a sparse dataset, reporting only what it found at the settings used; therefore, once the physical attributes for the 316 compounds were identified, a targeted analysis for all of these compounds was imposed on every sample. This resulted in a non-sparse dataset, i.e., there was a value for every sample for every compound. The metabolites were annotated by searching against an in-house metabolite database, Mass Spectrometry Metabolite Library of Standards (MSMLS) (http://iroa.com/page/Mass%20Spectrometry%20Metabolite%20Library%20of%20Standards). The dataset was then exported for analysis. The dataset was analyzed using the IROA ClusterFinder software. The peaks identified by ClusterFinder were compared against libraries of compounds for their IROA characteristics; C12 base peak, ^12^C M+1, ^13^C base peak, ^13^C M-1, and intervening peaks. The metabolic phenotype of each experimental sample consists of the relative concentration of a number of metabolites. In the IROA method, all compound measurements are made relative to a ^13^C standard; in this case, an isotopically-labeled wheat standard (97% ^13^C-enriched). Therefore, these measurements represent the deviation of each metabolite relative to the isotopically-labeled standard.

Data tables with metabolite peaks (mz/rt) at both control and high temperature stress conditions were formatted as comma separated values (.csv) files and uploaded to the MetaboAnalyst 3.0 server (http://www.metaboanalyst.ca) [38]. To improve the performance for downstream statistical analysis, metabolite data generated by LC-HRMS were checked for integrity and normalized using MetaboAnalyst’s normalization protocols (variables were mean-centered, Log2 transformed, and each variable was divided by its standard deviation (autoscaling). Univariate analyses were applied to calculate the fold change (FC) in metabolite concentrations between heat-stressed plants and control plants, and to assess statistical significance via t-test and one-way ANOVA. As the multivariate methods take all the variables into consideration, we applied multivariate method, Partial Least Squares Discriminant Analysis (PLS-DA), for comprehensive data analysis. The Supervised method, PLS-DA was used to maximize the difference of metabolic profiles between control and high temperature stress groups to enable the detection of metabolites existing in the biological samples [39]. Heat maps were created based on the Pearson distance measure and the Ward clustering algorithm, which displayed the top 25 features selected by partial least square discriminant analysis using a significance level of *P* ≤ 0.05, and Tukey’s HSD post-hoc analysis. For the metabolites that significantly changed with *P* < 0.05, pathway analysis was performed using MetaboAnalyst 3.0, web-based metabolomics data analysis software via Kyoto Encyclopedia of Genes and Genomes (KEGG) pathway database (http://www.genome.ad.jp/kegg/pathway.html) compared with *Oryza sativa* ssp. *japonica* (Rice Annotation Project Data Base http://rapdb.dna.affrc.go.jp) pathway library.

The six metabolites that showed the greatest increased or decreased FC under high temperature stress compared to control plants were selected for correlation analysis with physiology and yield measures in the R statistical software package. Pearson’s product-moment correlation coefficients were determined between metabolite concentration data and yield and physiological data for each pot across both temperature treatments. Significant correlations were identified at *P* < 0.05, *P* < 0.01 and *P* < 0.001.

Growth and yield data at final harvest were analyzed using analysis of variance procedures in the GLIMMIX procedure of SAS 9.4 (SAS Institute). The Gaussian conditional distribution and the identity link function were used. Residuals from each model fit were checked for normality graphically and numerically with the Shapiro-Wilk W test. Data were analyzed together across years because of similar results for each experiment, with genotype (CB202, CB205, CB208, CB210, CB213, and Ventnor) and temperature (24/18°C and 35/28°C) as fixed effects and year as a random effect. Degrees of freedom were determined using the Kenward-Roger method. Pairwise comparisons were made using the lsmeans statement with Tukey’s HSD method. Pearson’s product-moment correlation coefficients between metabolite concentration and different traits across all temperature and genotype data were estimated using SAS 9.4 (SAS Institute).

## Results

### Physiological parameters

**Fig 1:**
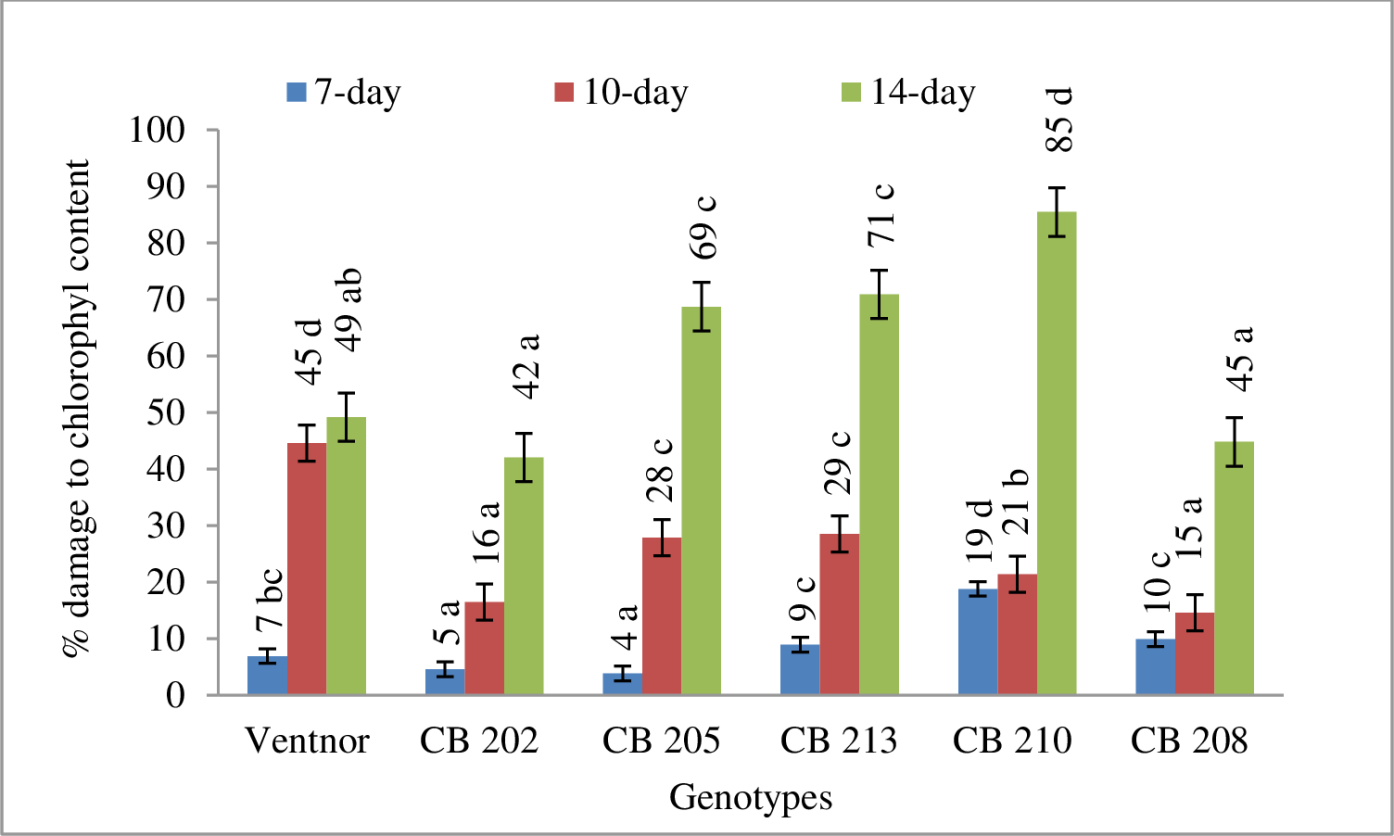
Damage to SPAD chlorophyll content (averaged across years) presented as percent (±SE) of control plants at 7-, 10-, 14-day after heat treatment. Bars representing mean values with same alphabets on the top are not significantly different (P<0.05) by Tukey's HSD test at a particular time of measurement.

**Fig 2:**
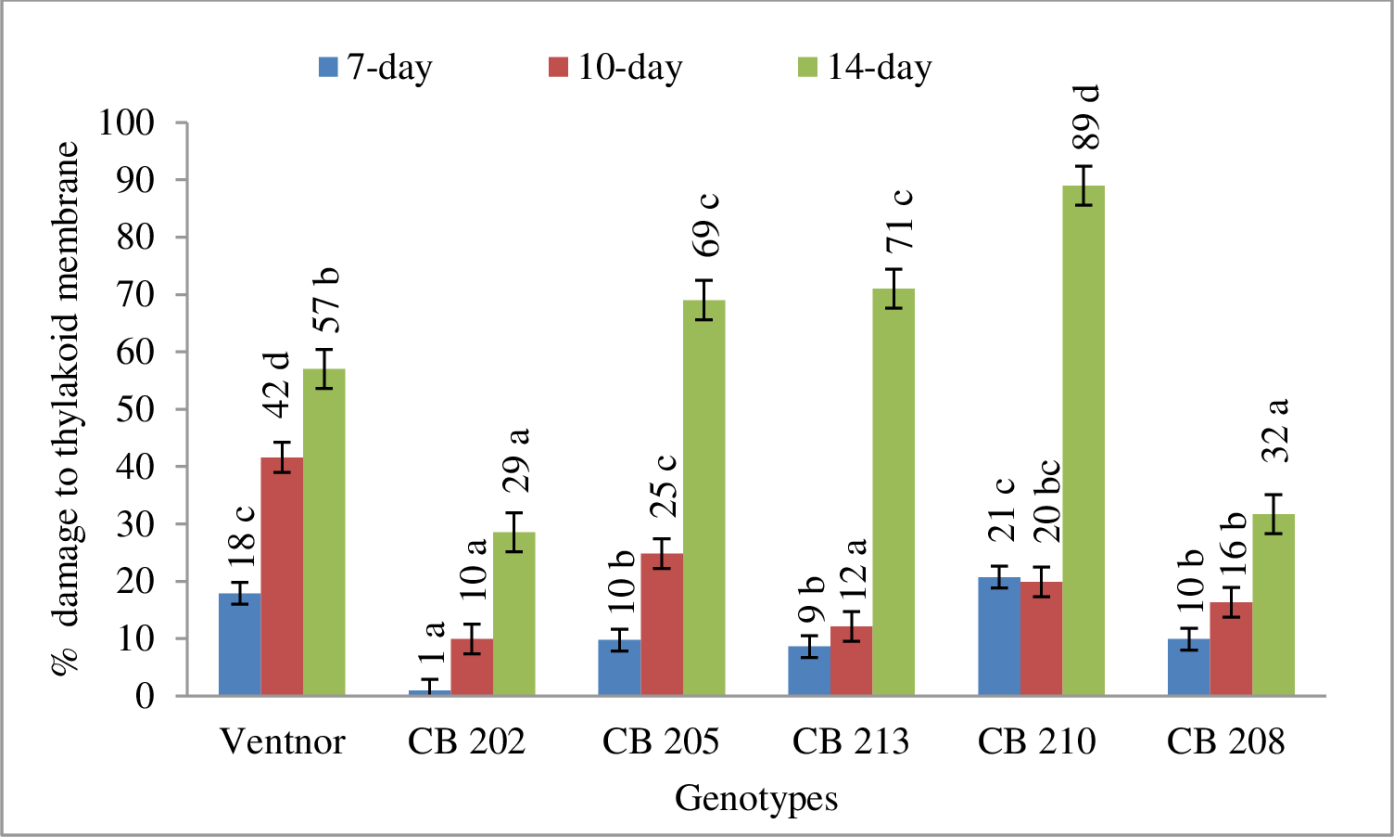
Damage to thylakoid membrane (averaged across years) presented as percent (±SE) of control plants at 7-, 10-, 14-day after heat treatment. Bars representing mean values with same alphabets on the top are not significantly different (P<0.05) by Tukey's HSD test at a particular time of measurement.

**Fig 3:**
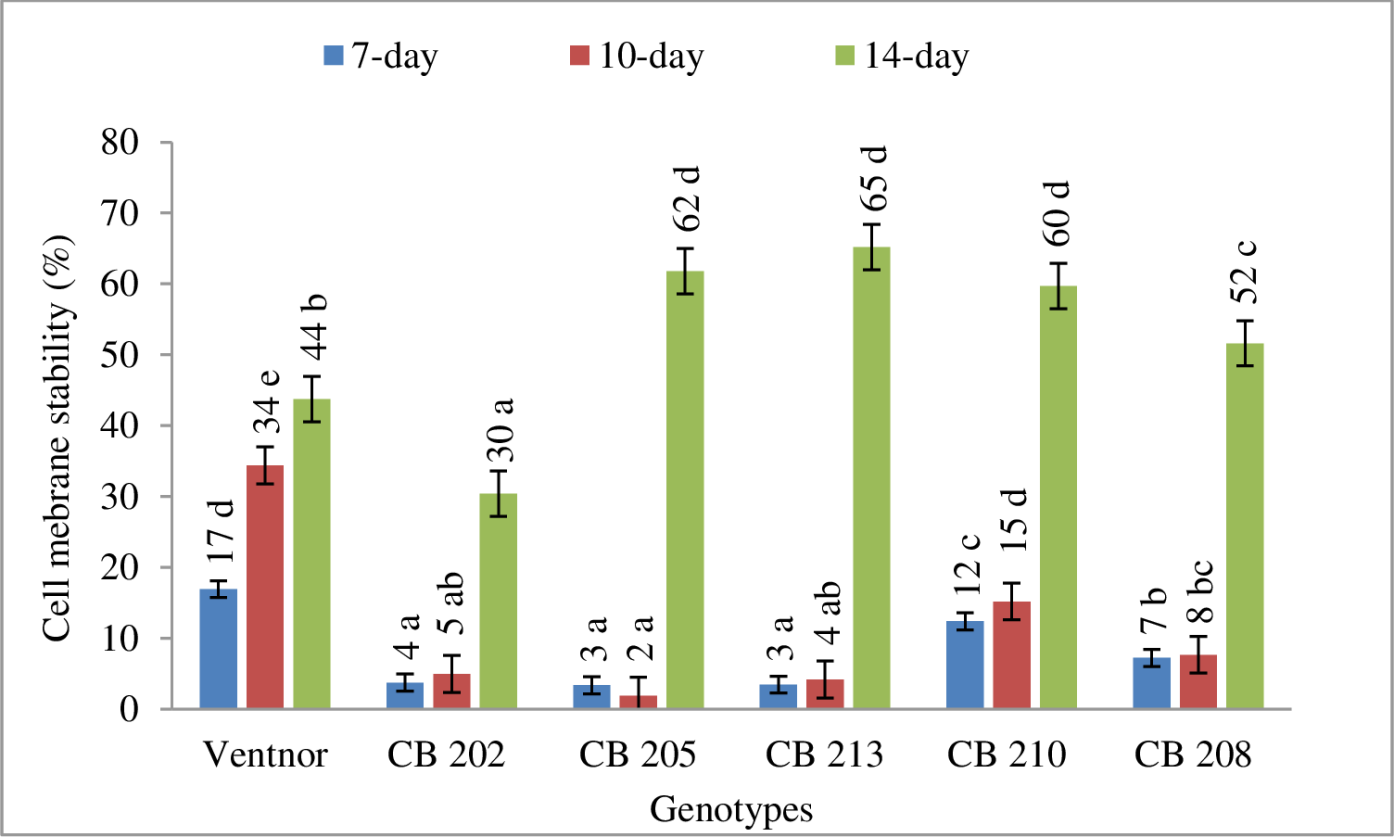
Cell membrane stability (averaged across years) presented as percent (±SE) of control plants at 7-, 10-, 14-day after heat treatment. Plasmamembrane damage was estimated at 7 and 14 days after heat stress in year 1, while at 7 and 10 days after heat treatment in year 2. Bars representing mean values with same alphabets on the top are not significantly different (P<0.05) by Tukey's HSD test at a particular time of measurement.

Table 1 is presenting the genetic effect on relative damage to SPAD chlorophyll content, chlorophyll fluorescence (F_V_/F_M_) and cell membrane stability due high temperature stress. The genotypic effect was significant for those physiological traits. The results indicated that all genotypes incurred damage to SPAD chlorophyll contact, F_V_/F_M_, and cell membrane stability due to high temperature stress. However, genotypes showed clear differences for relative damage to chlorophyll content, thylakoid and cell membrane due to high temperature (Figs 1, 2 and 3). CB202 and 208 showed least percent damage to those three traits due to high temperature and remained physiologically active for a longer amount of time. CB205, 213, and 210 demonstrated greatest damages for those traits under high temperature stress. These three genotypes showed a modest decline up to about 10 days for those traits and then a more drastic decline at 14 days (Figs 1, 2 and 3). Ventnor showed a steady decline for all those traits.

**Table 1:** Fixed effects ANOVA results for relative damage to SPAD chlorophyll content, F_V_/F_M_, cell membrane stability (CMS) due to temperature stress. The average values of three sampling dates across years were used for ANOVA.

Factor	SPAD Chlorophyll Content	F_V_/F_M_	Cell membrane stability
	F Ratio	F Ratio	F Ratio
Genotype	13.7**	6.3**	3.7*
Replication	4.6^NS^	2.6^NS^	3.4^NS^

*Significant at P < 0.05

** significant at P < 0.01; NS, not significant

### Yield related traits

**Fig 4:**
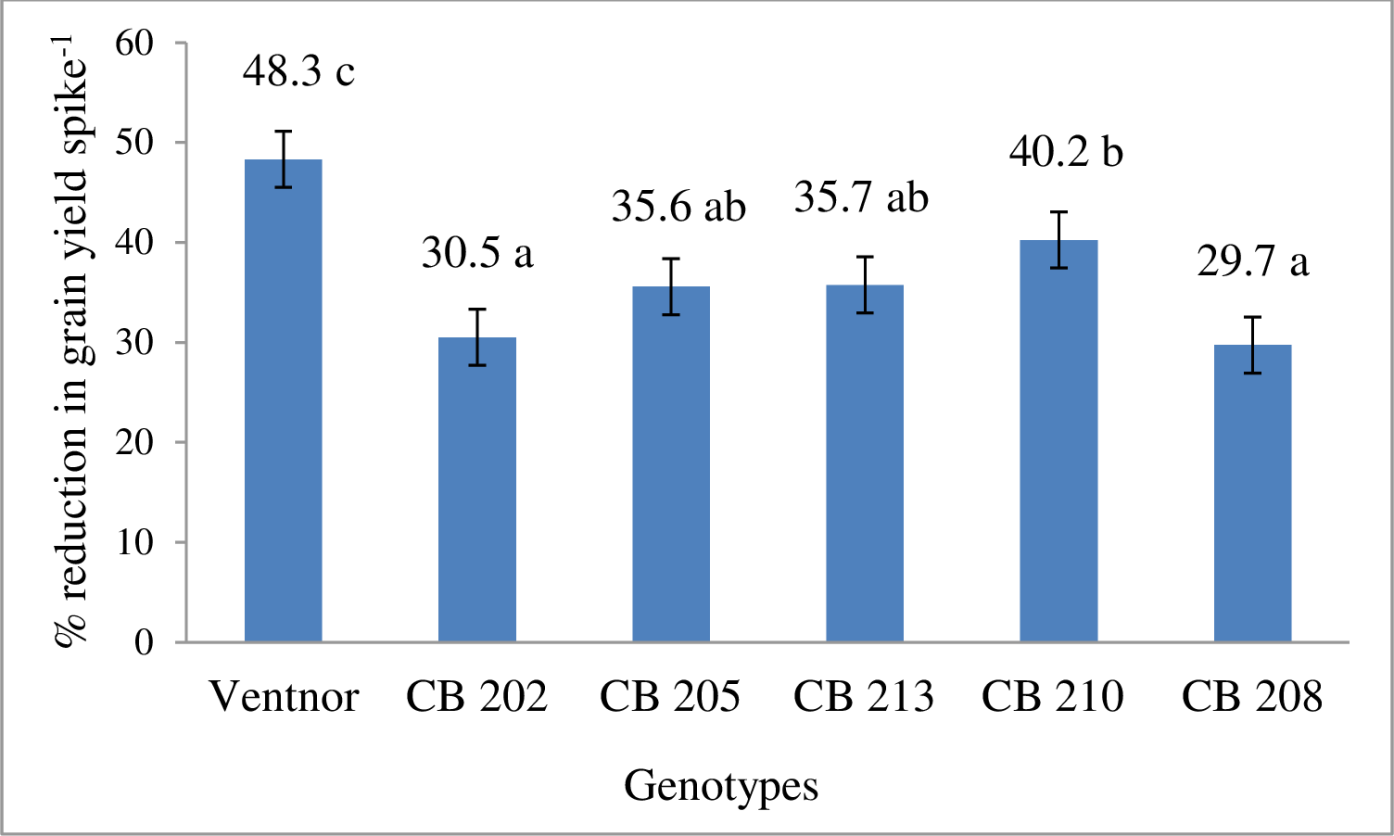
Reduction in grain yield per spike due to high temperature stress presented as percent (±SE) of control plants. Data combined across two years. Bars representing mean values with same alphabets on the top are not significantly different (P<0.05) by Tukey's HSD test.

**Fig 5:**
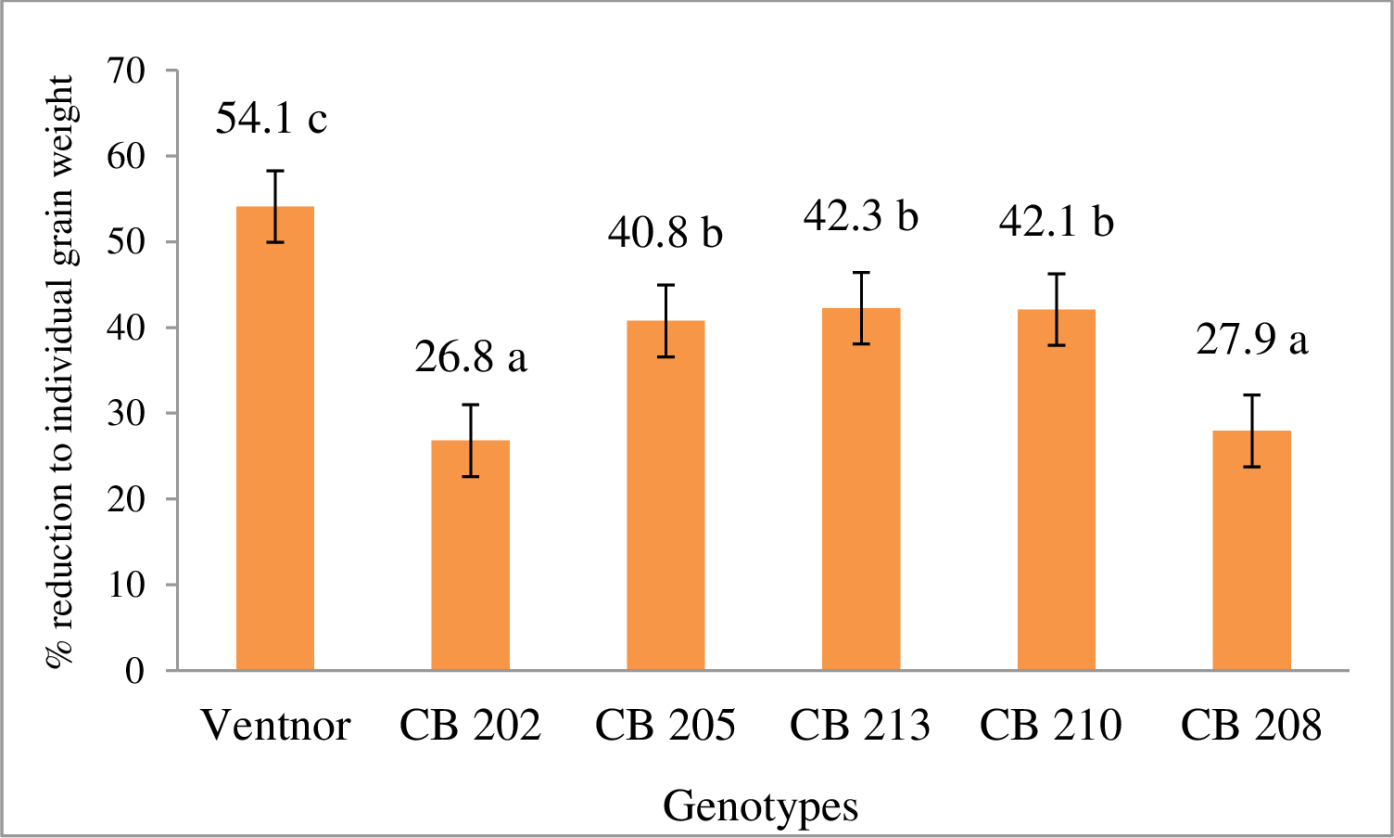
Reduction in individual grain weight due to high temperature stress presented as percent (±SE) of control plants. Data combined across two years. Bars representing mean values with same alphabets on the top are not significantly different (P<0.05) by Tukey's HSD test.

The genotypes showed significant effect for the reduction to grain yield per spike and individual grain weight due to high temperature stress (Table 2). However, genotypic effect was not significant for grains per spike reduction due to temperature stress. There were clear differences demonstrated by genotypes for relative reduction (in %) for grain yield per spike and individual grain weight due to high temperature stress (Figs 4 and 5). Percent reduction due to high temperature stress in grain yield per spike ranged from 29.7 to 48.3, while in individual grain weight ranged from 26.8 to 54.1. CB202 and 208 showed superior performances for minimizing high temperature stress effect on grain yield per spike and individual grain weight compare to other genotypes. Ventnor showed greatest reduction to those two traits due to high temperature stress. Percent reduction in grain yield per spike and individual grain weight to CB205, CB213, and CB210 was somewhat similar.

**Table 2:** Fixed effects ANOVA results for tiller number per plant, grain yield per spike, grains per spike, and individual grain weight. The average values across years were used for ANOVA.

Factor	Grain yield spike^-1^	Grains spike^-1^	Individual grain weight
	F Ratio	F Ratio	F Ratio
Genotype	6.3**	2.1^NS^	9.2***
Replication	0.5^NS^	0.4^NS^	1.8^NS^

*** significant at P < 0.001

NS, not significant

### Profiling of metabolites

**Fig 6:**
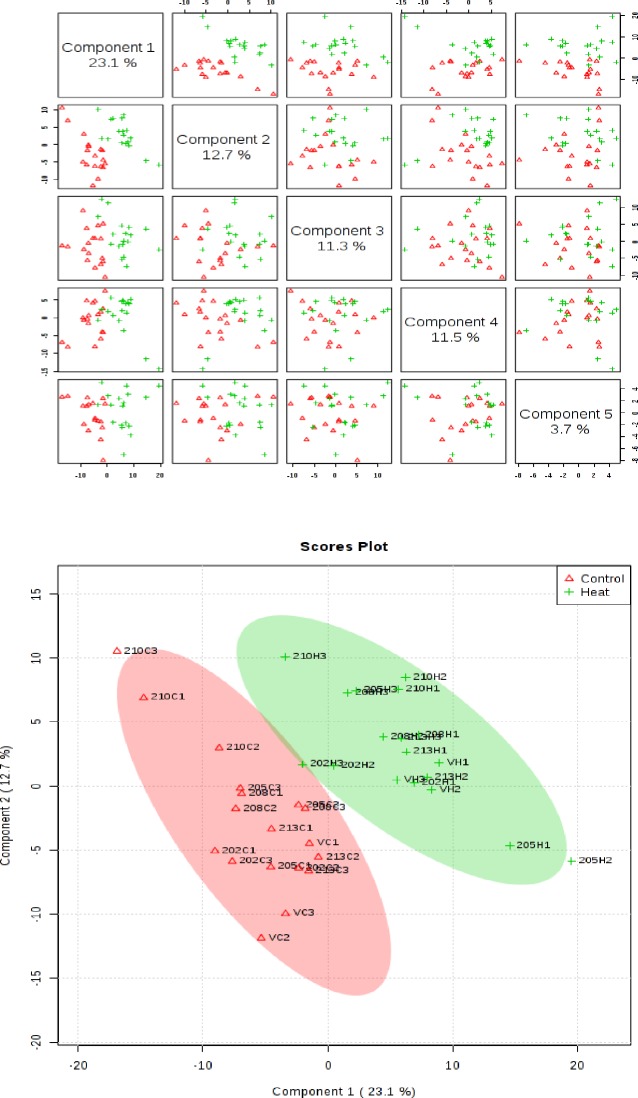
Partial least square discriminant analysis (PLS-DA) and 2D scores plot for the six wheat genotypes at mid grain filling under high temperature stress (H) and control condition (C). Metabolites at control and heat treatments didn’t overlap in general, indicating an altered state of metabolite levels in the wheat leaves. Treatments are thereby demonstrating effect in the leaves of wheat plants across different genotypes.

**Fig 7:**
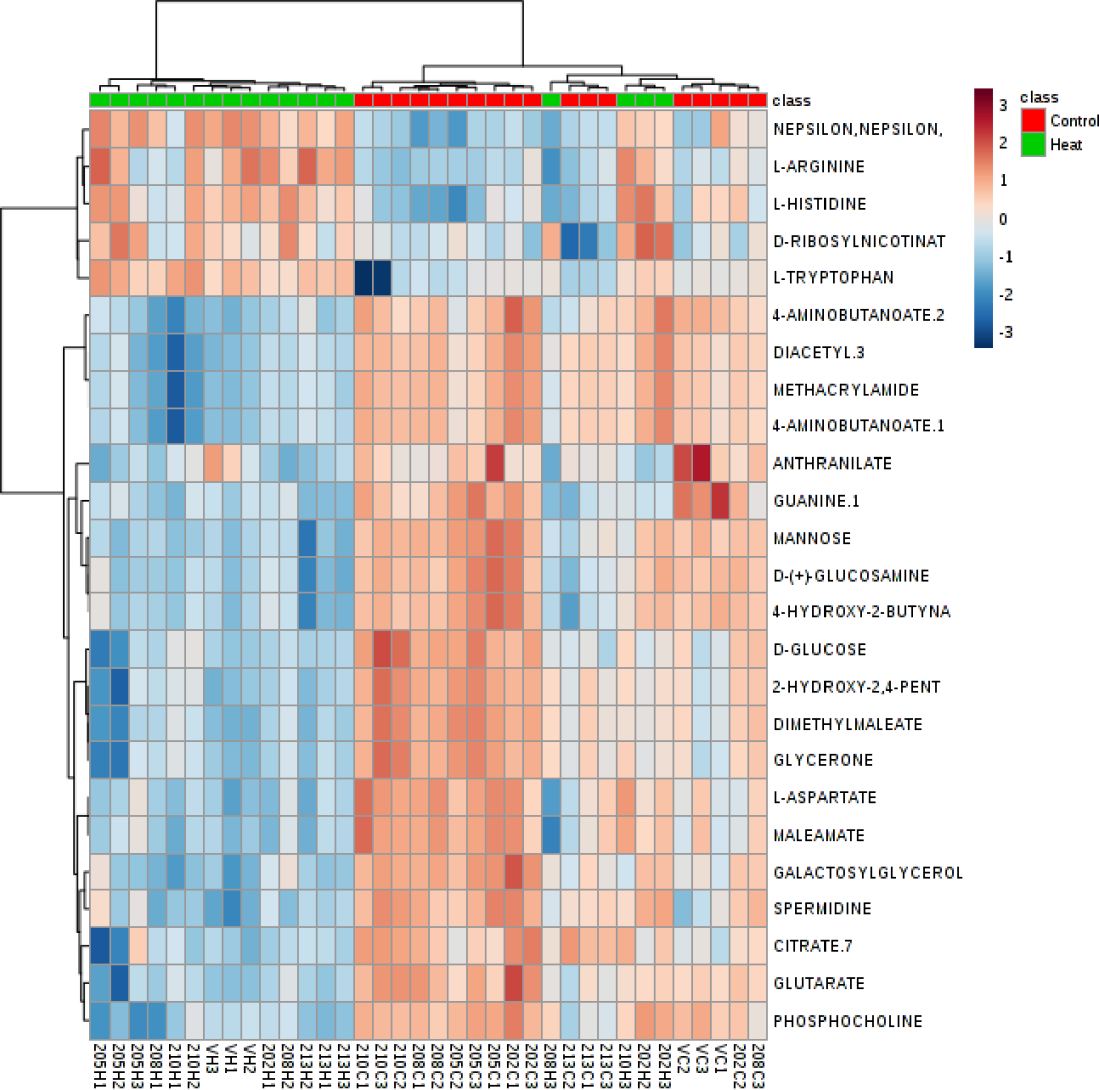
Hierarchical heat map is illustrating (distance measure: Pearson; Clustering algorithm: Ward) partial least square discriminant analysis (PLS-DA) showing levels of key metabolite. Metabolite feature areas were normalized and range-scaled across all experimental samples in a sub-set of metabolites found.

The metabolite profiling by LC-HRMS used in this study allowed a non-biased, untargeted global analysis and a total of 316 metabolites were identified. Of these 316 metabolites, 179 were identified as known compounds, 89 as possible compounds, and 48 metabolites were unknown. The supervised clustering method, Partial Least Squares-discriminant Analysis (PLS-DA) was performed for two treatments across genotypes (Fig 6) to condense the large set of correlated metabolite concentration data into a smaller set of linearly uncorrelated principal components. Using all variables, the PLS-DA explained a total of 62.3% of the total variation, with the first principal component (PC1) explaining 23.1% of the variance, while the second principal component (PC2) explained 12.7% of the variance across the dataset (Fig 6). The scores plot with PC1 and PC2 (Fig 6) revealed two distinct groups associated with heat-treated and control samples across the six genotypes. Thus, a clear distinction in metabolite expression due to temperature treatment was evident across all genotypes. The heat map generated from twenty-five metabolites differing between heat-stressed and control treatments showed similar clustering among genotypes (Fig 7). However, it also indicated some genotypic variability. For example, CB202 under heat stress was similar to other genotypes under heat stress for some metabolites like L-arginine and L-histidine, but was more similar to genotypes under control conditions for metabolites like 4-aminobutanoate.

**Fig 8:**
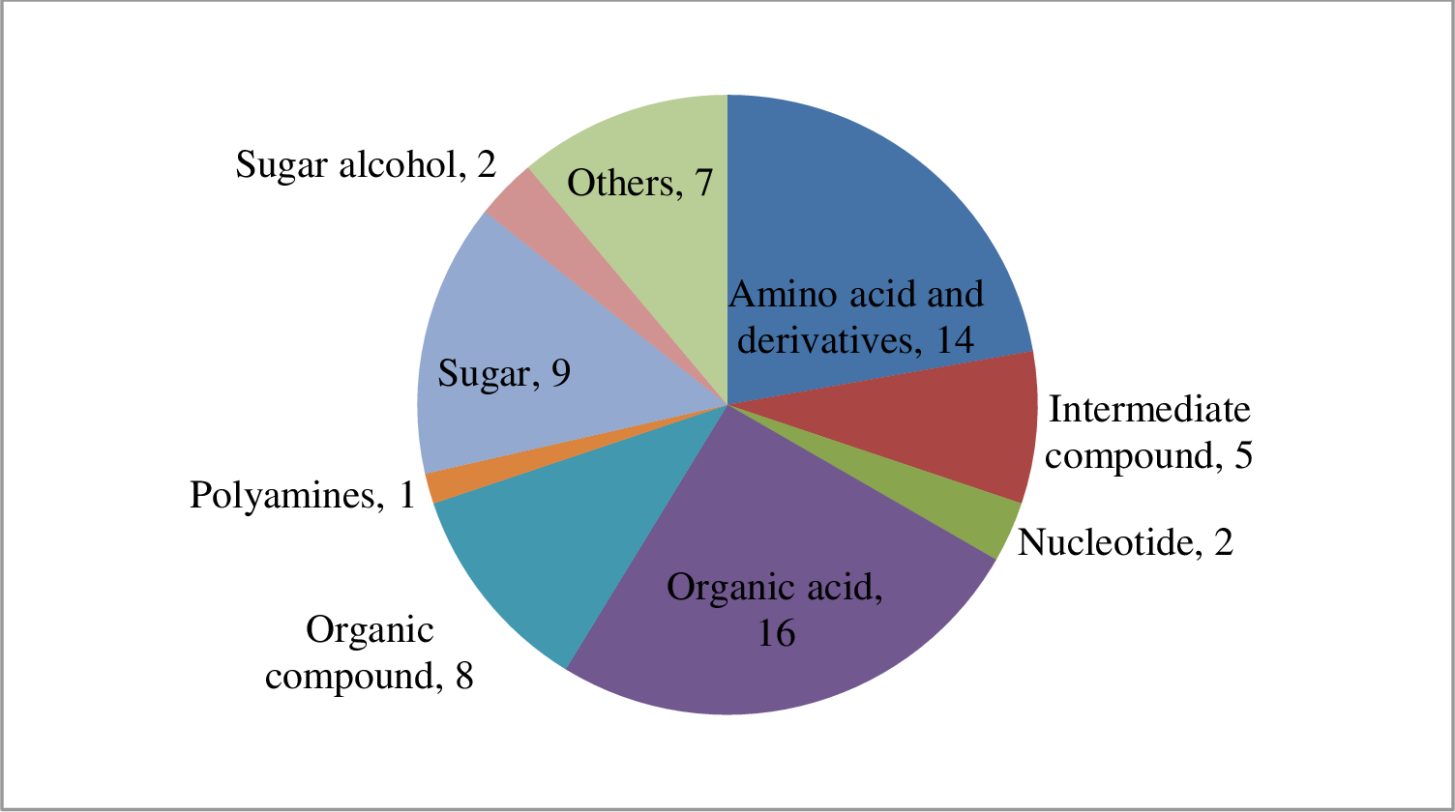
Diagram showing different classes of metabolites significantly accumulated under heat stress compare to controlled condition.

Further analysis of individual metabolites based on FC of metabolite concentration between heat-treated and control plants, indicated a total of 64 known metabolites that showed a significant change (>1.5 FC; *P* < 0.05; **Table A in S1 Text**), while 35 possible and unknown metabolites showed a significant change (>1.5 FC) and are presented in **Table B in S1 Text**. The sixty-four known metabolites were classified into different groups according to their chemical structures and published information (**Table A in S1 Text** and Fig 8). Fourteen amino acids and derivatives showed higher or lower accumulation due to heat treatment. For example, L-arginine, L-histidine, L-tryptophan, and leucine were all relatively high in heat-stressed plants compared to control plants, whereas amino acids like L-threonine, 4-aminobutanoate (GABA), L-aspartate, L-phenylalanine were found in lower concentrations in plants under heat stress. In general, organic acids declined under heat-stressed conditions compared with the respective controls, except for 3-hydroxypyruvic acid and 4-guanidinobutanoate, which increased considerably in heat-stressed plants. Organic compounds and sugar and sugar alcohols were two other major groups of metabolites that decreased in tissue concentration due to heat stress.

Although there was a difference in many metabolite levels between control and heat-stressed plants, further analyses focused on the metabolites that exhibited the greatest differences between the two treatments. This included L-tryptophan, pipecolate, alpha-aminoadipate, L-arginine, L-histidine, and piperidine, which showed the greatest increase in metabolite concentration in heat-stressed plants (Table 3). It also included drummondol, anthranilate, dimethylmaleate, galactoglycerol, guanine, and glycerone, which showed the greatest decrease in metabolite concentration in heat-stressed plants. These metabolites are involved in various biosynthesis/degradation pathways, including tryptophan, tyrosine and phenylalanine biosynthesis, biosysnthesis of secondary metabolites, lysine biosynthesis and degradation, glycine, serine, and threonine metabolism, aminoacyl-tRNA biosynthesis, Shikimate pathway, punier metabolism, glycerolipid metabolism, galactose metabolism, abscisic acid catabolism, and nicotinate and nicotinamide metabolism (Table 4). Metabolites that showed an increase in concentration in heat-stressed plants exhibited negative correlations with yield, SPAD, and F_V_/F_M_ (Tables 5 and 6), indicating that greater metabolite concentrations were associated with a greater reduction in yield, SPAD, and F_V_/F_M_. Metabolites that exhibited a decrease in metabolite concentration in heat-stressed plants were positively correlated with yield, SPAD, and F_V_/F_M_ (Tables 7 and 8), which meant that the plants that maintained higher levels of these metabolites had a greater yield, SPAD, and F_V_/F_M_.

**Table 3:** Fold change results for the top 12 metabolites showing the greatest increase or decrease in metabolite concentration in heat stressed plants compared to control plants.

Compounds	Fold change	*P*-value
L-tryptophan	11.46	<0.0001
Pipecolate	5.89	<0.001
Alpha-aminoadipate	8.57	<0.0001
L-arginine	3.68	<0.0001
L-histidine	3.36	<0.0001
Piperidine	2.15	<0.005
Drummondol	0.13	<0.0001
Anthranilate	0.19	<0.0001
Dimethylmaleate	0.31	<0.0001
Galactosylglycerol	0.32	<0.0001
Guanine	0.37	<0.0001
Glycerone	0.38	<0.0001

**Table 4:** Selected 12 metabolites and path way involved.

Compound	Pathway involved
L-Tryptophan	Aminoacyl-tRNA biosynthesis; glucosinolate biosynthesis; Phenylalanine, tyrosine and tryptophan biosynthesis; glycine, serine and threonine metabolism
Pipecolate	Lysine degradation; biosynthesis of secondary metabolites
Alpha-Aminoadipate	Lysine biosynthesis; lysine degradation; biosynthesis of secondary metabolites
L-Arginine	Aminoacyl-tRNA biosynthesis; arginine and proline metabolism
L-Histidine	Aminoacyl-tRNA biosynthesis; histidine metabolism
Piperidine	Tropane, piperidine and pyridine alkaloid biosynthesis; protein digestion and absorption
Drummondol	Abscisic acid catabolism
Anthranilate	Tryptophan metabolism; phenylalanine, tyrosine and tryptophan biosynthesis; biosynthesis of plant secondary metabolites; biosynthesis of alkaloids derived from shikimate pathway;
Dimethylmaleate	Nicotinate and nicotinamide metabolism
Galactosylglycerol	Galactose metabolism; glycerolipid metabolism
Guanine	Purine metabolism
Glycerone	Glycerolipid metabolism; methane metabolism

**Table 5:** Pearson’s product-moment correlation coefficients between metabolite concentration and kernel yield per pot, kernel yield per tiller, and average kernel weight across all temperature and genotype data. The top six metabolites showing the greatest increase in metabolite concentration in heat stressed plants compared to control plants were selected for correlation.

Compounds	Grain yield per spike	Individual kernel weight
	(g spike^1^)	(g kernel^-1^)
L-tryptophan	-0.55***	-0.46**
Pipecolate	-0.41*	-0.18
Alpha-aminoadipate	-0.67***	-0.38*
L-arginine	-0.72***	-0.55***
L-histidine	-0.72***	-0.60***
Piperidine	-0.66***	-0.46**

*Significant at P<0.05

**significant at P<0.01

***significant at P<0.001.

**Table 6:** Pearson’s product-moment correlation coefficients between metabolite concentration and SPAD, Fv/Fm and cell membrane stability across all temperature and genotype data. The top six metabolites showing the greatest increase in metabolite concentration in heat stressed plants compared to control plants were selected for correlation.

Compounds	SPAD	F_V_/F_M_	CMS
L-tryptophan	-0.45**	-0.48**	-0.50*
Pipecolate	-0.18	-0.17	0.79**
Alpha-aminoadipate	-0.30	-0.34*	0.08
L-arginine	-0.35*	-0.33	0.36*
L-histidine	-0.39*	-0.39*	-0.31
Piperidine	-0.22	-0.21	0.23

*Significant at P<0.05

**significant at P<0.01.

**Table 7:** Pearson’s product-moment correlation coefficients between metabolite concentration and kernel yield per pot, kernel yield per tiller, and average kernel weight across all temperature and genotype data. The top six metabolites showing the greatest decrease in metabolite concentration in heat stressed plants compared to control plants were selected for correlation.

Compounds	Grain yield per spike	Individual kernel weight
	(g spike^1^)	(g kernel^-1^)
Drummondol	0.08	0.25
Anthranilate	0.16	0.30
Dimethylmaleate	0.55***	0.66***
Galactosylglycerol	0.31	0.54***
Guanine	0.19	0.43**
Glycerone	0.14	0.18

**significant at P<0.01

***significant at P<0.001.

**Table 8:** Pearson’s product-moment correlation coefficients between metabolite concentration and SPAD and Fv/Fm across all temperature and genotype data. The top six metabolites showing the greatest decrease in metabolite concentration in heat stressed plants compared to control plants were selected for correlation.

compounds	SPAD	F_V_/F_M_	CMS
Drummondol	0.20	0.20	0.48**
Anthranilate	0.27	0.30	-0.28
Dimethylmaleate	0.57***	0.55***	0.54**
Galactosylglycerol	0.57***	0.57***	0.42*
Guanine	0.45**	0.43**	-0.58**
Glycerone	0.52**	0.50**	0.57**

*Significant at P<0.05

**significant at P<0.01

***significant at P<0.001.

## Discussion

High temperature stress is known to cause adverse effects on plant metabolic and physiological processes, and one of the most sensitive processes to abiotic stress is photosynthesis [40], which can lead to reduced yield and grain quality in wheat [41]. However, individual genotypes have shown differing abilities to maintain growth and productivity by acclimating to stress conditions through specific tolerance mechanisms. For instance, genotypic variability in different attributes related to photosynthesis and membrane structures are good indicators of stress tolerance [41] and yield [42]. Our results demonstrated a reduction in SPAD and F_V_/F_M_ in response to post-anthesis heat stress, and both years showed a similar trend among genotypes (Figs 1 and 2), which indicated that the heat treatment produced significant damage to the photochemistry in these genotypes. Previous study found that PSII sensitivity to high temperature in wheat was related to an increase in the fluidity of the thylakoid membrane [5]. Genotypes also showed significant cell membrane damage in this study. As genotypes showed some variability in the timing and severity of the photosystem and cellular membrane damage due to heat stress, correlations between this variability and metabolite accumulation can be used to further our understanding of the physiological basis for the observed metabolic variability in wheat, and can serve for future genetic improvement under post-anthesis heat stress conditions.

Heat induced yield reduction has been well documented in wheat. Elevated temperature during the flowering and grain filling stages reduces productivity [43]. Even small (e.g., 1.5°C) increases in temperature can have a negative effect on productivity. The optimum temperature regime during grain filling for wheat is 15–18°C [6; 44]. It has been estimated that every 1°C rise in temperature above the optimum temperature reduces yield per spike 3–4% [45], which is consistent with the maximum of 48% reduction in yield in the present study. Post-anthesis heat stress has minimal effect on kernel number [44; 46; 47], which was also observed in the present study. Average kernel weight showed a decrease at 35/28°C compared to 24/18°C, which was likely due to reduced grain filling duration from plants exposed to high temperature stress senescing before plants under optimal conditions [5]. In a similar study, kernel weight was decreased by 85% when the temperature rose from 20/16°C (day/night) to 36/31°C from 7 days after anthesis until maturity [9].

While a number of studies have examined the effects of post-anthesis high temperature stress on wheat yield and physiological parameters, information on associations between these phenotypic responses with altered states of different metabolites has not been examined thoroughly. We observed a number of metabolites that increased or decreased consistently across genotypes, which indicated that they were associated with the different biochemical pathways controlling high temperature stress tolerance in wheat. Arginine was among the metabolites identified that most strongly correlated with the yield and physiology data across all genotypes. Changes in amino acid levels of plants grown under stress conditions have been reported to be involved in different mechanisms, such as osmotic adjustment and detoxification of reactive oxygen species, and intracellular pH regulation [20; 48; 49]. Arginine is the biological precursor of nitric oxide which has been shown to play a role in plant senescence, by inducing or inhibiting it, and in hypersensitive responses to plant disease [50; 51]. Arginine had negative correlations with yield, SPAD, and F_V_/F_M_, which could indicate that greater accumulation of arginine results in the greater physiological damage and more yield loss. Therefore, it is possible that the role that nitric oxide played was to induce senescence or possibly the biosynthesis pathway to produce nitric oxide was inhibited and the nitric oxide could not inhibit senescence.

Alpha-aminoadipate and pipecolate had relatively strong negative correlations with yield and physiological parameters except CMS, which indicated that as the levels of these two metabolites increased, a greater reduction in yield was observed along with more damage to photosynthetic structures (Tables 7 and 8). Alpha-aminoadipate and pipecolate are catabolites of lysine, which is known to break down during plant stress into many metabolites [52; 53; 54]. Pipecolate has also been shown to be a strong osmolyte in the plant [52], and it also showed a strong positive correlation with CMS, which indicates that higher accumulation of this metabolite helped the wheat genotypes maintain normal cell membrane function. Tryptophan and histidine were also increased in heat stressed plants and negatively correlated to yield, SPAD, and Fv/Fm. Consistent with an increase in tryptophan under heat stress, anthranilate, a precursor to tryptophan, was found in lower concentrations in heat stressed plants [55]. Similar to their response to heat stress in the present study, pipecolate, tryptophan, and histidine were all increased in disease-stressed leaves [54].

Dimethylmaleate had some of the strongest positive correlations with yield, SPAD, and F_V_/F_M_. Dimethylmaleate is related to the TCA cycle and has been found to be co-localized with QTLs associated with agronomic traits including glaucousness, peduncle length, grains per m^2^, grain yield, and harvest index on chromosome 7A in the Excalibur/Kukri double haploid wheat population [56]. This metabolite showed a positive correlation with yield and other traits in wheat, and is also under genetic control, which indicates that it potentially plays an important function in plant high temperature tolerance and could be used for genetic improvement. Drummondol, one of the metabolites found that had a lower concentration in heat stressed plants than control plants, is a catabolite of abscisic acid [57]. This may suggest that there is more abscisic acid in heat stressed plants than in control plants. An increase in abscisic acid has been shown to protect plants from oxidative damage, have an increased relative water and chlorophyll content, which allow plants to better tolerate heat stress [58; 59].

## Conclusion

Our study demonstrated many of the metabolites that showed a large fold increase and were negatively correlated with yield, especially grain yield per spike, and physiology, such as tryptophan, arginine, and histidine, likely reflect this accelerated senescence, and might not necessarily be related to heat tolerance per se. Nevertheless, they could serve as potential biomarkers for breeding by selecting for those individuals that show a relatively lower accumulation of these metabolites, which could reflect delayed senescence (stay green) in these genotypes under heat-stressed conditions, and continue to produce photosynthetic assimilates for developing grain. In contrast, metabolites that were found in lower concentrations in heat-stressed plants, especially dimethylmaleate and galactosylglycerol, were positively correlated with average kernel weight and physiological function. Relatively little is known about these metabolites and further research is needed to better understand their role in plant metabolism and response to heat stress. However, they could also serve as potential biomarkers for breeding by selecting for those individuals that exhibit relatively high concentrations of these metabolites. Our study identified metabolites with differential accumulation under control and post-anthesis heat-stressed conditions, and the associations between different phenotypic traits known to vary with heat stress and metabolites have been established. The robustness of metabolic change and their relationship with phenotypes renders those as good candidates for bio-marker assisted breeding. Although some of these newly identified metabolites offer promise as biomarkers for improving high temperature stress tolerance in wheat, genetic dissection for metabolic pathways involved with those metabolites require further investigation. Further research will need to be done to confirm that these metabolites are consistently found under heat stress conditions and are consistently increased or decreased in heat stressed plants under field conditions.

## Supporting information

S1 Text Table A. Fold change (FC), p-value, false discovery rate (FDR), Kyoto Encyclopedia of Genes and Genomes (KEGG ID), PubChem ID (*), molecular formula (MF), mass-to-charge ratio (m/z), and retention time (RT) of metabolites identified as significant in the study. Table B. Fold change (FC) p-value, false discovery rate (FDR), molecular formula (MF), mass-to-charge ratio (m/z), and retention time (RT) of possible/unknown metabolites identified as significant in the study.
(DOCX)
