# Supplementary material for: Comparative physiological and metabolomics analysis of wheat (Triticum aestivum L.) following post-anthesis heat stress
Source: PLoS One. 2018 Jun 13;13(6):e0197919. doi: 10.1371/journal.pone.0197919 (PMC5999278; doi:10.1371/journal.pone.0197919)
Supplement: S1 Text — Table A. Fold change (FC), p-value, false discovery rate (FDR), Kyoto Encyclopedia of Genes and Genomes (KEGG ID), PubChem ID (*), molecular formula (MF), mass-to-charge ratio (m/z), and retention time (RT) of metabolites identified as significant in the study. Table B. Fold change (FC) p-value, false discovery rate (FDR), molecular formula (MF), mass-to-charge ratio (m/z), and retention time (RT) of possible/unknown metabolites identified as significant in the study. (DOCX) [file pone.0197919.s001.docx]

### Table A. Fold change (FC), p-value, false discovery rate (FDR), Kyoto Encyclopedia of Genes and Genomes (KEGG ID), PubChem ID (*), molecular formula (MF), mass-to-charge ratio (m/z), and retention time (RT) of metabolites identified as significant in the study

| **Compound name** | **Compound type** | **FC** | **p-value** | **FDR** | **KEGG/**  **PubChem** | **MF** | **m/z** | **RT** |
| --- | --- | --- | --- | --- | --- | --- | --- | --- |
| L-tryptophan | Amino acid and derivatives | 11.46 | 0.0000151 | 0.000181 | C00806 | C_11_H_12_N_2_O_2_ | 205.097 | 7.67 |
| L-arginine | Amino acid and derivatives | 3.68 | 0.0000169 | 0.000197 | C00062 | C_6_H_14_N_4_O_2_ | 175.119 | 0.72 |
| L-histidine | Amino acid and derivatives | 3.36 | 0.0000189 | 0.000209 | C00135 | C_6_H_9_N_3_O_2_ | 156.077 | 0.72 |
| Leucine | Amino acid and derivatives | 2.02 | 0.0065991 | 0.032847 | C16439 | C_6_H_13_NO_2_ | 132.103 | 2.17 |
| N(Alpha)-Acetyl-L-Arginine | Amino acid and derivatives | 0.66 | 0.0001868 | 0.001609 | 40521* | C_8_H_16_N_4_O_3_ | 217.130 | 1.62 |
| L-threonine | Amino acid and derivatives | 0.59 | 0.0005030 | 0.003653 | C00188 | C_4_H_9_NO_3_ | 118.051 | 0.72 |
| N-hydroxy-l-valine | Amino acid and derivatives | 0.55 | 0.0001068 | 0.001007 | C20313 | C_5_H_11_NO_3_ | 134.081 | 0.76 |
| L-serine | Amino acid and derivatives | 0.54 | 0.0076613 | 0.036106 | C00716 | C_3_H_7_NO_3_ | 106.050 | 0.72 |
| L-phenylalanine | Amino acid and derivatives | 0.49 | 0.0044421 | 0.024294 | C02057 | C_9_H_11_NO_2_ | 166.087 | 5.12 |
| 4-aminobutanoate.2 | Amino acid and derivatives | 0.49 | 0.0000082 | 0.000122 | C00334 | C_4_H_9_NO_2_ | 104.071 | 0.09 |
| 4-aminobutanoate.1 | Amino acid and derivatives | 0.42 | 0.0000047 | 0.000078 | C00334 | C_4_H_9_NO_2_ | 104.071 | 0.09 |
| L-phenylalanine.1 | Amino acid and derivatives | 0.47 | 0.0013196 | 0.008352 | C02057 | C_9_H_11_NO_2_ | 166.087 | 5.66 |
| L-aspartate | Amino acid and derivatives | 0.46 | 0.0000021 | 0.000045 | C16433 | C_4_H_7_NO_4_ | 134.045 | 0.73 |
| Anthranilate | Amino acid and derivatives | 0.19 | 0.0000099 | 0.000136 | C00108 | C_7_H_7_NO_2_ | 138.055 | 0.9 |
| Alpha-aminoadipate | Intermediate compound | 8.57 | 0.0000378 | 0.000399 | C00956 | C_6_H_11_NO_4_ | 162.076 | 0.89 |
| Phosphocholine | Intermediate compound | 0.39 | 0.0000240 | 0.000259 | C00588 | C_5_H_14_NO_4_P | 184.074 | 0.73 |
| Drummondol | Intermediate compound | 0.13 | 0.0000689 | 0.000663 | 6440575* | C_13_H_20_O_4_ | 239.129 | 11.16 |
| Oxaloglutarate.2 | Intermediate compound | 0.58 | 0.0012164 | 0.007924 | C05533 | C_7_H_8_O_7_ | 203.020 | 1.71 |
| Oxaloglutarate.1 | Intermediate compound | 0.41 | 0.0061170 | 0.031510 | C05533 | C_7_H_8_O_7_ | 203.019 | 1.83 |
| Adenine | Nucleotide | 0.39 | 0.0084647 | 0.038658 | C00147 | C_5_H_5_N_5_ | 134.047 | 1.52 |
| Guanine.1 | Nucleotide | 0.37 | 0.0000004 | 0.000012 | C00242 | C_5_H_5_N_5_O | 152.058 | 6.22 |
| Pipecolate | Organic acid | 5.89 | 0.0016765 | 0.010461 | C00408 | C_6_H_11_NO_2_ | 130.087 | 1.2 |
| 3-hydroxypyruvic acid | Organic acid | 1.57 | 0.0138500 | 0.026812 | C00168 | C_3_H_4_O_4_ | 103.003 | 1.13 |
| 4-guanidinobutanoate.1 | Organic acid | 1.53 | 0.0158800 | 0.023378 | C01035 | C_5_H_11_N_3_O_2_ | 146.094 | 1.42 |
| Cis-aconitate.3 | Organic acid | 0.66 | 0.0012624 | 0.008105 | C00417 | C_6_H_6_O_6_ | 173.009 | 2.35 |
| Glucarate | Organic acid | 0.65 | 0.0002878 | 0.002199 | C00767 | C_6_H_10_O_8_ | 209.030 | 0.77 |
| (S)-malate.2 | Organic acid | 0.60 | 0.0175050 | 0.037431 | C00149 | C_4_H_6_O_5_ | 133.014 | 1.03 |
| 3-methyl-2-oxobutanoate | Organic acid | 0.60 | 0.0131880 | 0.025117 | C00141 | C_5_H_8_O_3_ | 117.055 | 0.86 |
| Cis-aconitate.9 | Organic acid | 0.52 | 0.0005709 | 0.004079 | C00417 | C_6_H_6_O_6_ | 173.009 | 2.76 |
| Citrate.7 | Organic acid | 0.51 | 0.0000184 | 0.000209 | C00158 | C_6_H_8_O_7_ | 191.020 | 8.04 |
| N-acetyl-l-glutamate.1 | Organic acid | 0.49 | 0.0002143 | 0.001791 | C00624 | C_7_H_11_NO_5_ | 188.057 | 2.2 |
| Maleamate | Organic acid | 0.46 | 0.0000057 | 0.000087 | C01596 | C_4_H_5_NO_3_ | 116.035 | 0.73 |
| Quinate | Organic acid | 0.43 | 0.0025787 | 0.014836 | C00296 | C_7_H_12_O_6_ | 191.056 | 0.82 |
| Glutarate | Organic acid | 0.41 | 0.0000003 | 0.000012 | C00489 | C_5_H_8_O_4_ | 131.035 | 1.79 |
| Glycerate | Organic acid | 0.41 | 0.0002511 | 0.001986 | C00258 | C_3_H_6_O_4_ | 105.019 | 0.82 |
| Traumatic acid | Organic acid | 0.41 | 0.0043171 | 0.024209 | C16308 | C_12_H_20_O_4_ | 227.129 | 10.75 |
| Dimethyl maleate | Organic acid | 0.31 | 0.0000000 | 0.000003 | C00922 | C_6_H_8_O_4_ | 143.035 | 0.76 |
| Piperidine | Organic compound | 2.15 | 0.0056216 | 0.029647 | 18049* | C_5_H_11_N | 86.097 | 2.15 |
| L-alpha-glycerophosphocholine | Organic compound | 1.72 | 0.0497120 | 0.148700 | 55397* | C_8_H_20_NO_6_P | 258.110 | 0.74 |
| Glycerophosphoglycerol | Organic compound | 0.53 | 0.0009509 | 0.006582 | C03274 | C_6_H_15_O_8_P | 245.043 | 0.76 |
| 1,2,3-trihydroxybenzene.3 | Organic compound | 0.52 | 0.0000415 | 0.000418 | C01108 | C_6_H_6_O_3_ | 127.039 | 0.08 |
| 2,3-dihydroxybenzoate | Organic compound | 0.47 | 0.0011504 | 0.007722 | C00196 | C_7_H_6_O_4_ | 153.019 | 8.88 |
| Diacetyl.3 | Organic compound | 0.41 | 0.0000043 | 0.000074 | C00741 | C_4_H_6_O_2_ | 85.029 | 3.18 |
| Glycerone | Organic compound | 0.38 | 0.0000010 | 0.000025 | C00184 | C_3_H_6_O_3_ | 89.024 | 0.78 |
| Galactosylglycerol | Organic compound | 0.32 | 0.0000017 | 0.000041 | C05401 | C_9_H_18_O_8_ | 255.108 | 0.83 |
| Spermidine | Polyamines | 0.40 | 0.0000442 | 0.000435 | C00315 | C_7_H_19_N_3_ | 146.165 | 0.6 |
| D-ribose | Sugar | 1.78 | 0.0004590 | 0.003389 | C00121 | C_5_H_10_O_5_ | 149.046 | 0.91 |
| Glycerone.2 | Sugar | 0.65 | 0.0073707 | 0.035781 | C00184 | C_3_H_6_O_3_ | 89.024 | 1.2 |
| Lichenin.4 | Sugar | 0.64 | 0.0145360 | 0.028539 | C00478 | C_6_H_10_O_5_ | 161.045 | 1.14 |
| D-glucose.2 | Sugar | 0.63 | 0.0169780 | 0.036561 | C00031 | C_6_H_12_O_6_ | 179.056 | 1.1 |
| Lichenin | Sugar | 0.63 | 0.0074308 | 0.035781 | C00478 | C_6_H_10_O_5_ | 161.045 | 0.9 |
| Lichenin.1 | Sugar | 0.57 | 0.0001889 | 0.001609 | C00478 | C_6_H_10_O_5_ | 161.045 | 0.91 |
| D-(+)-glucosamine | Sugar | 0.48 | 0.0000035 | 0.000065 | C00329 | C_6_H_13_NO_5_ | 180.087 | 0.74 |
| Mannose | Sugar | 0.42 | 0.0000001 | 0.000005 | C00159 | C_6_H_12_O_6_ | 203.053 | 0.76 |
| D-glucose | Sugar | 0.39 | 0.0000007 | 0.000019 | C00031 | C_6_H_12_O_6_ | 179.056 | 0.8 |
| D-arabinono-1,4-lactone | Sugar alcohol | 1.58 | 0.0003232 | 0.002427 | C00652 | C_5_H_8_O_5_ | 147.030 | 1.51 |
| D-glucono-1,5-lactone | Sugar alcohol | 0.65 | 0.0002464 | 0.001985 | C00198 | C_6_H_10_O_6_ | 177.040 | 0.74 |
| Nepsilon,nepsilon,nepsilon-trimethyllysine | Others | 2.36 | 0.0000008 | 0.000023 | C03793 | C_9_H_20_N_2_O_2_ | 189.160 | 0.72 |
| D-ribosylnicotinate.2 | Others | 1.59 | 0.0000051 | 0.000081 | 58527* | C_11_H_13_NO_6_ | 256.082 | 1.03 |
| Tert-butoxycarbonyl anhydride | Others | 0.62 | 0.0250780 | 0.032451 | 48500* | C_10_H_18_O_5_ | 217.108 | 8.73 |
| Ethylammonium propionate | Others | 0.53 | 0.0029983 | 0.017029 | 63884* | C_5_H_13_NO_2_ | 120.102 | 0.76 |
| 4-hydroxy-2-butynal.1 | Others | 0.51 | 0.0000151 | 0.000181 | C02648 | C_4_H_4_O_2_ | 85.029 | 0.89 |
| Methacrylamide | Others | 0.40 | 0.0000026 | 0.000053 | 51759* | C_4_H_7_N_O_ | 86.061 | 0.79 |
| 2-hydroxy-2,4-pentadienoate | Others | 0.36 | 0.0000003 | 0.000012 | C00596 | C_5_H_6_O_3_ | 113.024 | 0.77 |

### Table B. Fold change (FC) p-value, false discovery rate (FDR), molecular formula (MF), mass-to-charge ratio (m/z), and retention time (RT) of possible/unknown metabolites identified as significant in the study

| **compound name** | **FC** | **p-value** | **FDR** | **MF** | **m/z** | **RT** |
| --- | --- | --- | --- | --- | --- | --- |
| Possibly C11H23NO6 | 0.232 | 0.0000001 | 0.000004 | C_11_H_23_NO_6_ | 266.160 | 0.82 |
| Possibly C13H20N6O7P | 4.075 | 0.0423940 | 0.133200 | C_13_H_20_N_6_O_7_P | 196.044 | 7.47 |
| Possibly C15H21N12O4 | 0.257 | 0.0000148 | 0.000181 | C_15_H_21_N_12_O_4_ | 434.187 | 0.83 |
| Possibly C23H21N9O5 | 0.243 | 0.0011173 | 0.007615 | C_23_H_21_N_9_O_5_ | 502.157 | 8.13 |
| Possibly C34H43N16O3 | 0.575 | 0.0293870 | 0.101710 | C_34_H_43_N_16_O_3_ | 722.370 | 12.49 |
| Possibly C6H14N3OS2 | 0.359 | 0.0000000 | 0.000003 | C_6_H_14_N_3_OS_2_ | 207.051 | 0.85 |
| Possibly C6H14N3OS2.1 | 0.639 | 0.0057186 | 0.029804 | C_6_H_14_N_3_OS_2_ | 207.051 | 0.93 |
| Possibly C6H6N2O7 | 0.469 | 0.0000033 | 0.000064 | C_6_H_6_N_2_O_7_ | 219.027 | 0.72 |
| Possibly C6H7N3O11 | 2.523 | 0.0001566 | 0.001387 | C_6_H_7_N_3_O_11_ | 296.002 | 0.73 |
| Possibly C6H9N4O3P | 0.320 | 0.0000004 | 0.000012 | C_6_H_9_N_4_O_3_P | 215.033 | 0.76 |
| Possibly C6H9N5O6P | 0.352 | 0.0001466 | 0.001325 | C_6_H_9_N_5_O_6_P | 277.023 | 0.77 |
| Possibly C6H9N5O7P | 0.588 | 0.0389600 | 0.126910 | C_6_H_9_N_5_O_7_P | 293.018 | 0.77 |
| Possibly C7H12N5O4 | 3.497 | 0.0000041 | 0.000072 | C_7_H_12_N_5_O_4_ | 231.098 | 1.98 |
| Possibly C7H7N5O3P | 1.519 | 0.0136790 | 0.056634 | C_7_H_7_N_5_O_3_P | 241.038 | 1.06 |
| Possibly C7H8N2O8 | 0.343 | 0.0000001 | 0.000007 | C_7_H_8_N_2_O_8_ | 249.037 | 0.72 |
| Possibly C8H17N6O6P2 | 2.067 | 0.0453890 | 0.139630 | C_8_H_17_N_6_O_6_P_2_ | 354.063 | 0.77 |
| Possibly C8H17NO11 | 2.764 | 0.0002348 | 0.001926 | C_8_H_17_NO_11_ | 302.073 | 0.73 |
| Possibly C9H14N3O6 | 1.844 | 0.0000001 | 0.000004 | C_9_H_14_N_3_O_6_ | 261.095 | 1.87 |
| Possibly C9H14N3O7 | 0.347 | 0.0000019 | 0.000042 | C_9_H_14_N_3_O_7_ | 277.089 | 0.84 |
| Possibly C9H14N7O5 | 0.282 | 0.0000000 | 0.000003 | C_9_H_14_N_7_O_5_ | 299.098 | 0.85 |
| Possibly C9H15N5O8P | 0.301 | 0.0000093 | 0.000132 | C_9_H_15_N_5_O_8_P | 351.060 | 0.85 |
| UNK MZ 109.1243 RT 0.81 | 0.609 | 0.0000396 | 0.000408 | Not available | 104.108 | 0.81 |
| UNK MZ 136.0436 RT 0.74 | 0.590 | 0.0002677 | 0.002081 | Not available | 129.019 | 0.75 |
| UNK MZ 148.0610 RT 0.75 | 0.478 | 0.0000116 | 0.000156 | Not available | 145.050 | 0.76 |
| UNK MZ 151.0698 RT 1.07 | 2.817 | 0.0160620 | 0.063529 | Not available | 148.061 | 1.07 |
| UNK MZ 188.0321 RT 0.77 | 1.870 | 0.0172460 | 0.067017 | Not available | 185.022 | 0.77 |
| UNK MZ 204.1176 RT 0.74 | 0.440 | 0.0000018 | 0.000042 | Not available | 198.097 | 0.74 |
| UNK MZ 219.0954 RT 0.71 | 2.205 | 0.0017994 | 0.010959 | Not available | 213.075 | 0.71 |
| UNK MZ 223.0495 RT 0.76 | 0.314 | 0.0000004 | 0.000013 | Not available | 217.030 | 0.76 |
| UNK MZ 224.0631 RT 0.71 | 0.473 | 0.0001316 | 0.001215 | Not available | 219.048 | 0.72 |
| UNK MZ 231.0819 RT 0.72 | 0.288 | 0.0000000 | 0.000003 | Not available | 225.062 | 0.73 |
| UNK MZ 231.2195 RT 0.79 | 0.556 | 0.0000137 | 0.000179 | Not available | 221.186 | 0.79 |
| UNK MZ 283.0532 RT 0.75 | 0.194 | 0.0000000 | 0.000002 | Not available | 242.052 | 0.75 |
| UNK MZ 327.1177 RT 0.77 | 5.222 | 0.0401290 | 0.128470 | Not available | 319.091 | 0.77 |
| UNK MZ 535.2733 RT 0.81 | 0.200 | 0.0106340 | 0.045736 | Not available | 520.224 | 0.8 |
